# Strategies for the Prevention of Violence in Institutional Care: A Qualitative Interview Study With Ward Managers

**DOI:** 10.3389/fpsyt.2022.853260

**Published:** 2022-04-08

**Authors:** Martin Lindow, Lars Kjellin, Ingemar Engström, Veikko Pelto-Piri

**Affiliations:** University Health Care Research Center, Faculty of Medicine and Health, Örebro University, Örebro, Sweden

**Keywords:** violence, values, coercive measures, prevention, residential homes, adolescents, ward manager, qualitative content analysis

## Abstract

**Background:**

The presence of violence within psychiatric and residential settings remains a challenge. Research on this problem has often focused on describing violence prevention strategies from either staffs' or service users' perspectives, and the views of ward managers has been largely overlooked. The aim of the present study was therefore to identify ward managers' strategies to prevent violence in institutional care, focusing on safety and values.

**Methods:**

Data were collected using semi-structured interviews with 12 ward managers who headed four different types of psychiatric wards and two special residential homes for adolescents. Qualitative content analysis was applied, first using a deductive approach, in which quotes were selected within a frame of primary, secondary, and tertiary prevention, then by coding using an inductive approach to create themes and subthemes.

**Results:**

Ward managers' strategies were divided into the four following themes: (1) *Balancing being an active manager with relying on staff's abilities* to carry out their work properly while staying mostly in the background; (2) *Promoting value awareness and non-coercive practices in encounters with service users* by promoting key values and adopting de-escalation techniques, as well as focusing on staff-service user relationships; (3) *Acknowledging and strengthening staff's abilities and competence* by viewing and treating staff as a critical resource for good care; and (4) *Providing information and support to staff* by exchanging information and debriefing them after violent incidents.

**Conclusions:**

Ward managers described ethical challenges surrounding violence and coercive measures. These were often described as practical problems, so there seems to be a need for a development of higher ethical awareness based on a common understanding regarding central ethical values to be respected in coercive care. The ward managers seem to have a high awareness of de-escalation and the work with secondary prevention, however, there is a need to develop the work with primary and tertiary prevention. The service user group or user organizations were not considered as resources in violence prevention, so there is a need to ensure that all stakeholders are active in the process of creating violence prevention strategies.

## Introduction

Psychiatric and special residential care differs from somatic care, not only with respect to the nature of the care given, but also by the institutions' legal rights to use coercion of service users. There are many special prerequisites in institutions where coercive measures may be used in daily care that give rise to a range of problems and ethical dilemmas. We use “institutional care” as a collective term for institutions, like psychiatric inpatient care or special residential homes, where staff have the legal right to use coercion. We believe that despite differences between these institutions, there are also great similarities, they have a “coercive context” which presents similar problems for managers and staff to deal with (1, 2). One such problem is the parallel treatment of a heterogeneous group of service users with different diagnoses, co-morbidities and social problems (3). Another challenge within these kind of institutions is the presence of interpersonal violence in daily life on the ward, which can lead to coercive measures against service users and cause more violence and injuries to anyone involved. This issue has been the focus of many studies, and from different perspectives (4–8). One study has found that most mental health care staff members at psychiatric wards in Sweden have at some point been subject to violence; of these, almost half had experienced violence within the last 6 months (9). Violence is also common in special residential care and a major problem for staff (10). Despite efforts made to prevent violence, the number of violent incidents in Swedish special residential homes has been increasing (11). Staff exposure to violence can cause anxiety, fear and sleeping problems, it can also result in losing confidence in, and perceiving a value incongruence with, the management (6, 9). So, it is emotionally difficult to work in an environment where violence occurs frequently and it influences the way staff members negotiate their work identity (12). Staff members need to regulate their emotions in relation to their colleagues, the “feeling rules” at the ward, but also toward themselves and the service users. Especially young people can be mocking with each other, “playfight” without the purpose of creating a serious conflict (13). In these cases, there is a risk that staff, due to their emotions, overreact and thus create a conflict that can lead to physical violence and coercive measures (14, 15). In Sweden, as well as in other European countries, much work has been dedicated to reducing the occurrence of violence and creating institutional care environments that are safe (16–19). Despite this, caregivers that work within different forms of institutional care still run a daily risk of being subject to both verbal and physical violence (6, 8). The damages arising from the occurrence of violence within institutional care also generate substantial costs related to the provision of safe care for service users, as well as costs of sickness absence for staff (4).

Preventive strategies in health care are traditionally divided into three distinct levels, as follows: primary, secondary, and tertiary prevention (20–22). The role of primary prevention is to prevent disease incidence. Placing a focus on preventing the occurrence of violence implies working at the organizational level and identifying risk factors, according to a widely adopted model in six domains; staff team, physical environment, outside hospital, service user community, service user characteristics, and regulatory framework (23). Interventions at this level are, for instance, physical security efforts like functioning alarms, but also soft security, an environment that is positive and promotes a friendly communication style (20, 23, 24). Secondary prevention includes early detection and targeted intervention through identification of situations or service users that may involve an increased risk of conflicts (23). Recommended interventions are to use calm down methods, de-escalation techniques and pro re nata medication (16, 23, 25, 26). Creating a relationship to the service users and to plan daily routines and activities in a way that would minimize escalating situations is often highlighted by staff as an important aspects of work, it helps them to recognize problems in an early stage and also to have an idea of what measures that need to be taken in the event of a conflict (7, 10). Tertiary prevention involves actions that aim to reduce harm by minimizing injuries during violent incidents, taking care of those involved in such incidents such that they do not suffer psychological or physical harm, and preventing future incidents from reoccurring. Common models often include a focus correctly using coercive measures such as forced medication, seclusion, or physical restraint (10, 16). Follow-up of incidents include the use of debriefing in special cases, but also staff support groups allowing the team to reflect on their experiences and reactions to their work with service users (27). The management can monitor and evaluate whether objectives of violence prevention have been achieved and facilitate actions with the aim of improving current practices (20). In this improvement work, service users' stories can provide valuable information (28).

Ward managers' “management style” has been identified as crucial when it comes to preventing the occurrence of violence in psychiatric care settings. They are, together with senior management, responsible for the prevention work (20, 29). Jakobsson et al. (29) has suggests that ward managers' strategies in health care “could include risk assessments, prevention, evaluation, education and reflection combined with, for example, scenario training”. They emphasize that violence in somatic care is most often relatively harmless, which is not in line with experience from psychiatric inpatient care and special residential homes (1–15). This means that ward managers in psychiatric and special residential homes need to work more proactively; creating a user-friendly environment and help staff to early detect risky situations and service users. Successful leadership strategies include supporting staff and securing their continuous training, working with guidelines, and functioning as a role model by setting examples of good behavior, while also ensuring good working conditions (30–32). Key values of this management style include an emphasis on communication and dialogue, with “open door” policy, as well as adopting interpersonal positive attitudes and respect (20). An essential part of prevention in health care is the training of staff that can consist of raising the awareness and understanding of violence, to recognize and assess potential risk situations and learn to use adequate intervention (20). The most common training in Swedish institutions for dealing with violence is a course, called TERMA, Bergen model (in psychiatry) or No Power No Lose (in special residential homes). These courses normally contain two parts. First a theory part on violence; how to understand violent behavior and emotional reaction to it, legislation, ethics and de-escalation. The second part is skills training on de-escalation and physical techniques, including coercive measures. The Bergen model is based on existing research (33) and the most evidence-based of these. TERMA and No Power, No Lose have gradually been supplemented with research on violence, mainly on de-escalation techniques.

Concerns have been raised about discrepancies between the use of coercive measures and compliance with human rights, such as the Convention Against Torture and Other Cruel, Inhuman or Degrading Treatment or Punishment (34) and the Declaration on the Rights of Disabled Persons (35). A great deal of effort has been invested in mapping and reducing the occurrence of coercive measures to create psychiatric care environments that are both safe and promote actual care, while simultaneously fully respecting human rights (18, 36–39). Several promising interventions to prevent violence and coercive measures in institutional care have been developed, but policies that make full use of these interventions are still missing (18, 23, 36). There is a continuing need for the promotion of non-coercive strategies for dealing with service users, such as developing communication skills and caring attitudes among staff, as well as facilitating the formation of staff-service user relationships through various violence prevention programs like Safewards, Six Core Strategies and Trauma-Informed Care intervention (18, 23, 25, 33, 36, 38). The ability of ward managers to facilitate the implementation of programs and guidelines is somewhat limited, they need education and training to develop leadership skills and strategies to plan and support implementation (39). The World Health Organization recommends that strategies at all levels of prevention should be developed together with all stakeholders (20), implying values such as (1) reciprocity in relations, to acknowledge the service user as an expert on his/her illness, and work accordingly to the service user's values and needs, and (2) social justice, to develop care and treatment processes together with service users and other stakeholders in society, and improve staff's clinical practice skills (24, 40).

Service users and staff alike have the right to spend their days and nights in an environment in which they feel safe and secure. This may be accomplished by different physical arrangements, but also needs to be based on professional ethical values that constantly guide staff-service user interactions. A key person in establishing and maintaining such values is the manager of the ward. The ward manager's task is difficult, especially when it comes to balancing administrative work with the quality of front line clinical care. Despite this, many ward managers seem to feel confident about their own ability to support staff, but they need support from the senior management (41, 42). Extensive research efforts have been invested in creating safer care environments, some of which have described strategies to deal with or prevent violence, from either staff's or service users' point of view (5, 6, 43). The current empirical research on preventive strategies in institutional care has, however, largely overlooked the perspective of ward managers. The aim of the present study was thus to identify the strategies used by ward managers to prevent violence in institutional care, focusing on safety and values.

## Materials and Methods

### Design

Data were collected using semi-structured interviews with ward managers, and were subject to qualitative content analysis (44, 45). All interviews were conducted as part of the ongoing research project “Prevention of violence in institutional care, aspects of ethics and safety”. The aim of the project was to study how institutions balance the need for security with ethics in encounters with service users. The study was approved by the Ethical Review Board, Uppsala, Sweden, reference number 2014/112.

### Settings and Sample

The setting consisted of different types of wards within institutional care. The six included wards were in one general psychiatric clinic, one medium security forensic psychiatric clinic, one psychiatric addiction center, one child and adolescent psychiatric clinic, and two special residential homes for adolescents, operated by The National Board of Institutional Care. One of these special residential homes was for adolescent girls mainly under 15 years old, and the other was for adolescent boys mainly over 15 years old. The population at the special residential homes consists of adolescents with complicated psychosocial problems, most often in connection with mental disorders and/or neuropsychiatric developmental disturbances. The forensic psychiatric clinic and special residential homes provided only coercive care, whereas the others provided a mix of voluntary and coercive care. The general psychiatric and psychiatric addiction clinics cared for all patients in need of psychiatric inpatient care from their region, the forensic clinic and the special residential homes had service users from the whole of Sweden. Substance users could be found in all participating institutions, but the psychiatric addiction clinic was specialized to care for patients with a combination of addiction and psychiatric diagnoses. All institutions we wanted to include in the study chose to participate. All of them, except the psychiatric addiction center, participating in this study utilized some form of 2-day training as described in the introduction. However, the level of ambition varied greatly between the different clinics, the forensic clinic had the highest ambition with mandatory training for all staff members.

The sample for this study was chosen in purpose to gain as many perspectives on violence prevention strategies as possible. The inclusion criteria were institutions, as different as possible, where staff have a legal possibility to use coercion toward service users. During the project, there was a complete change of ward managers. Both the outgoing and the incoming managers of these wards were interviewed, resulting in a total sample of 12 ward managers (7 men and 5 women), seven managers had more than 10 years of experience of institutional care and four had at least 6 years. One of the managers was new to psychiatric inpatient care, he chose to do the interview together with the outgoing manager who had long experience. At least half of the managers had a University degree in a health care profession, predominantly in nursing. The others had previously made a manager career in the health care sector or other services like the prison service and the Swedish armed forces. We also interviewed staff and service users at the same institutions to obtain all stakeholders' perspectives on violence prevention, which have been presented in previous scientific publications (5–7, 9, 15, 46).

### Interview Guide and Procedure

The semi-structured interview guide included the following question areas: (1) values at work, especially regarding interactions with the service users, (2) general safety questions, and (3) concrete handling of critical incidents. The interview guide was based on our and others previous research on ethics, violence and coercion and it was not pilot tested, the interview guide is available, see (47).

We first informed the ward mangers *via* email about our research project and its aim, and invited them to participate. We informed the ward managers verbally that the interview predominantly concerned violence in institutions, with a focus on questions concerning ethics and safety. The ward managers were interviewed at their respective workplace by one main interviewer and one observer who could ask supplementary questions. All interviews were completed in a single session, and were recorded and transcribed ad verbatim, only the participants and researchers were present, field notes were not used. VP participated in most of the interviews, mostly as an observer, but also as the main interviewer. The ward mangers did not have a previous relationship to the researchers. The interviews lasted for about 1–1.5 h and the transcribed material amounted to ~20 pages of written text per interview. The transcripts were not returned to participants for comment or correction. Preliminary findings from the project were discussed at three seminars with some of the participating wards.

### Analysis and Interpretation

The transcribed interviews were subjected to a qualitative content analysis. This analysis began with a deductive approach, whereby relevant quotes were selected and placed within the categories of primary, secondary, and tertiary prevention. The quotes were coded and thereafter categorized in higher-order headings using an inductive approach (44, 45). Themes could be related to several levels of prevention, whereas subthemes were most often related to one level of prevention.

Relevant sentences were coded within the different prevention categories. VP, IE, and LK each made suggestions for themes/categories within one prevention category. ML coded four interviews by himself and suggested themes, thereafter comparing his codes and themes with previous coding and thematization of the other researchers. Themes were created first without any hierarchical order, later on ML and VP established a preliminary framework for themes and sub-themes which was changed during the analysis. All coding and thematization done by one of the researchers were then checked by, and discussed with, at least one other researcher. All decisions throughout the data analysis process were taken with consensus (48). The final framework of themes and subthemes was finalized in a dialogue with all authors. Our understanding of the field came from the authors' professional education (medicine, social science and pedagogy), experience of psychiatric services, and review of the literature. Information on the background of the authors can be found in Author Contributions.

## Results

The results are presented as four different themes (Table 1), which were as follows:

- *Balancing being an active manager with relying on staff's abilities*. The manager trusted staff to carry out their work properly while staying mostly in the background, although they were at hand when needed.
- *Promoting value awareness and non-coercive practices in encounters with service users*. The manager worked actively on staff-service user relationships while promoting key values and the use of de-escalation techniques.
- *Acknowledging and strengthening staff's abilities and competence*. The manager viewed staff as a critical resource whose competence and commitment to their work was a determining factor for good care.
- *Providing information and support to staff*. In this way, the ward manager facilitated information exchange through various forums and organized debriefings after violent incidents.

**Table 1:** Themes and subthemes identified on different levels of prevention.

**Themes**	**Subthemes**
Balancing between being an active manager with relying on staff's abilities	Trusting staff to follow guidelines^a^ Creating a sense of “us”^a^ Siding with staff^c^
Promoting value awareness and non-coercive practices in encounters with service users	Maintaining a continuous discussion about common values^a^ Strengthening staff-service user relationships through staff presence^a^ Advocating the use of de-escalation techniques and communication about critical incidents^b^, ^c^
Acknowledging and strengthening staff's abilities and competence	Employing the right person and creating incentives for commitment to work^a^ Raising staff level of competence regarding safety and handling of violent incidents^a^ Ensuring sufficient staffing levels in violent situations^a^, ^b^
Providing information and support to staff	Enabling meetings for information and support^a^ Organizing debriefings^c^

a *Primary prevention*,

b *Secondary prevention, and*

c *Tertiary prevention*.

Quotations that illustrated the strategies of ward managers are provided under each theme and were associated with different levels of prevention.

### Balancing Being an Active Manager With Relying on Staff's Abilities

#### Trusting Staff to Follow Guidelines

A recurring strategy among ward managers was attempting to create a balance between being sometimes present and sometimes absent from the ward. Several managers described how important it was for them to be visible and supportive; at the same time, there was a clear ambition to remain in the background as much as possible, and to have confidence that their staff would do a good job. In taking a step back, managers needed to trust that staff would follow guidelines and make well-informed decisions. Several ward managers considered written guidelines to be an important tool for staff, but noted that, to be practically useful, these documents need to be kept up to date.

*My goal is for it [the guidelines] to become a living document, it should become like a handbook for work in the ward. One shouldn't have to hesitate, but simply grab a file and open it, “okay so this is what we decided, and this is what we should do”, it should be clear to everyone*. (Manager 4, residential home)

Ward managers who made use of this strategy seemed to have a personal leadership style that was characterized by having trust in their staff's abilities. The ambition of being a visible leader and being available was a frequent theme among several ward managers, and recurred in several different ways throughout the interviews. One of the managers said that their door was always open for members of the staff if an incident occurred, and made sure that staff were aware of this.

#### Creating a Sense of “us”

Managers also expressed an ambition to encourage all staff members to strive toward the same goal and that it was important to create a sense of “us”. By working with the staff in this manner, being available when needed and otherwise staying in the background, many of the ward managers described how staff came together as a group and felt confident in making independent decisions in accordance with both written guidelines and their ethical convictions.

*But I try to do that, bring everyone onboard. To know why we do it and what we do, even though we sometimes don't decide anything we must know why and what it is, and I will know more about the team, which I think helps them too. Also, if something happens, they will know who to turn to. A more visible leader is always better*. (Manager 2, general psychiatric clinic)

To promote staff confidence, ward managers gave positive feedback to staff when any violent or otherwise difficult situation was resolved successfully. Some ward managers especially sought to give praise in concrete situations, which was not always possible to do due to the fact that the ward manager simply was not present on the floor and therefore would not detect such situations at all. Consequently, there was a sense that violent situations and behaviors often slipped under the ward manager's radar despite efforts to the contrary.

*I would like to make it concrete when staff are doing good things. At the same time, my position today as ward manager keeps me from being very close to staff in their daily work […], and that's when you wish that, when being in a ward environment and you witness a concrete event that is very good, you can be there to encourage and to say “well done”*. (Manager 2, child and adolescent psychiatric clinic)

#### Siding With Staff

Ward managers who had trust in staff's abilities also recognized the importance of trusting staff's judgment and siding with staff in critical situations. This meant that, when there was an incident, the assessment of the staff present at the scene would determine if the alarm should or should not be sounded. This kind of view served a double purpose of both strengthening staff's confidence, and making sure that staff could stay one step ahead of violent incidents and thereby preventing them from taking place.

*I have also said that the one who assesses or observes the patient must be taken seriously, that there are no false alarms in that sense*. (Manager 2, general psychiatric clinic)

Several managers expressed the opinion that one condition for a safe care environment is that staff never doubt their manager's confidence in their decisions. By trusting staff to make good decisions in a somewhat unsafe environment, staff would inevitably need to stay alert and self-aware of their own actions, which also served to take some burden off the ward manager's shoulder in terms of leading the work on the ward. This faith in staff also stretched from colleague to colleague, forming a bond of trust and mutual responsibility that allowed staff to act more decisively as a group.

*Let's say that a staff member is going to see to a youngster, and we don't really know the state of the person, then we always go together. So, we always make an assessment as to whether there needs to be more of us. Sometimes there's even been four of us!* (Manager 3, residential home)

### Promoting Value Awareness and Non-coercive Practices in Encounters With Service Users

#### Maintaining a Continuous Discussion About Common Values

Ward managers expressed an awareness of the importance of values and ethics within institutional care. Service users that act violently or make verbal threats were viewed with compassion, and managers expressed their wish to help these service users in the best possible manner. Such approaches call for common values that are shared among staff and permeate the care given, and this hinges on continuous discussions. Several managers reported the need to keep value issues alive through discussions in various forums, and reported attempting to create a standard by setting a good example and leading staff onto the right track when necessary. Furthermore, some ward managers had the sense that they were mainly responsible for preparing the mindset of newly recruited staff. New employees often needed a lot of support to be able to carry out their work because it was difficult to recruit competent staff.

*I believe that since I cannot choose, and always have to take the ones who seek my ward, then it is my responsibility to really define what it is that we are doing and what my expectations are, as well as what the employee's expectations are*. (Manager 2, child and adolescent psychiatric clinic)

Discussions about values and ethics was something that many managers regarded as important factors for giving meaning to the work being carried out in the ward; many also considered this to be a process that would optimally arise from the bottom-up rather than coming from above.

#### Strengthening Staff-Service User Relationships Through Staff Presence

From the perspective of primary prevention, managers stressed the importance of service user's sense that staff were present and available, and that good relationships between service users and staff were established, which enhanced the care given. Furthermore, ward managers strived to instill in staff a sense of working in the ward primarily for the benefit of the service users, thereby requiring them to actively commit themselves to the task. This was most evident from managers' reminders to staff to not confine themselves to offices or spend too long on computers, and to instead be present among the ward's service users. Spending time with service users and working proactively with violence prevention were also highlighted as important factors that contributed to improving the mood of service users.

*We need to be very alert and attentive to what is happening and to the mood of the youths. So, it's really important that we are close to the group at all times, so that we can see what is happening and feel what is happening*. (Manager 4, residential home)

Several managers reported that creating good staff-service user relationships constituted the foundation of actual violence prevention, rather than physical factors. Several managers emphasized that a staff member who has established a sufficiently stable relationship with the service user will also be able to set limits for that service user. This became especially clear within the care for children and adolescents, where relationship-making was pointed out as a key to violence prevention, a condition that was underscored by many examples of situations that had gotten out of control due to lack of relation to, and knowledge of, the service user.

*And then there were some that could actually handle conflicts, talk them down, manage to be physical, and were able to set limits in a fair way. Some who had enough alliances, enough good relationships, who had won enough social capital with the youth to actually be able to give reprimands in a nice way. Or in a tough way*. (Manager 2, residential home)

#### Advocating the Use of De-escalation Techniques and Communication About Critical Incidents

Another way of working with secondary violence prevention that came to light was the promotion of the use of de-escalation techniques by staff and the application of certain models, such as the Bergen model, to facilitate the formation of staff-service user relationships. Several managers reported making an effort to actively set limits and react to different kinds of unacceptable behavior that frequently occurred. Particularly within child and adolescent psychiatric care, social skills training was regarded as an important tool to teach service users what constitutes acceptable behavior and what kind of behaviors do not work in society. At the same time, managers admitted that staff's levels of tolerance were often very high, and that more experienced staff in particular tended to let verbal or physical violence pass them by without too much concern, which, according to ward managers, posed a problem.

*Your level of tolerance increases when working with this, and you accept comments such as “I'll stick this pencil in your eye”. You accept that it's just something that he says, rather than telling him that kind of behavior won't work in society. So, the problem is that the level of tolerance is slowly raised. So, we try to communicate that this behavior is not acceptable*. (Manager 2, forensic psychiatric clinic)

Verbal threats and aggressiveness can be signs of imminent physical violence, a fact obvious to most ward managers. This was another strong reason for suppressing such tendencies at an early stage. Among several ward managers, there was a strong awareness that a certain kind of behavior was not acceptable either within a health care setting or in society in general, and there was a subsequent sense of duty to guide service users by reacting to such behavior. Within this awareness, there was also conflict, since reacting according to the rules for every minor incident would be an immense waste of time and resources, which could negatively affect care. Additionally, some ward managers shared stories about incidents that they had reported to the police, only to later find that it had led to no legal consequences whatsoever. The purpose of such a procedure was therefore questioned.

Several of the managers believed that after a violent incident had taken place, staff should somehow give feedback to the service user concerned, perhaps by sitting down together and having a follow-up conversation, as a kind of debriefing. This kind of tertiary prevention procedure was also something that, according to written regulations, should take place, yet it did not happen routinely.

*We actually don't have it as a routine. I can't say that we do. That if a patient has been very violent, that you should sit down together afterwards. Sometimes things fall out that way, you sit down together in peace and quietness, but I can't say that things always fall out that way*. (Manager 1, psychiatric addiction center)

Giving feedback to the service user in other kinds of situations and discussing unacceptable behaviors was reported by ward managers as being important for two reasons. First, for the sake of the service user, as they were then given the opportunity to change their behavior, and second, for the benefit of the staff, since the service user's view of how staff reacted could offer valuable insights when working with violence prevention. By actively taking part in this feedback cycle, the ward managers sought to promote an environment where dialogue was considered normative.

### Acknowledging and Strengthening Staff Qualities and Resources

#### Employing the Right Person and Creating Incentives for Commitment to Work

An important part of ward managers' strategies for preventing violence rested on staff as a critical resource. Several managers emphasized the value of ensuring that the right kind of people are involved in the work, and that this aspect posed a management challenge. When searching for suitable staff, some recurring criteria were personal suitability, sense of responsibility, and ethical awareness. While this kind of informal competence was highly sought after by several ward managers, they often needed to prioritize formal competence. Some managers, however, looked mainly at personal characteristics as grounds for employment, since such a big part of care is based on establishing good staff-service user relationships. Also, the low educational level of some staff, when speaking of violence prevention, would incline certain ward managers to look mainly at other personal qualities as grounds for employment.

*When we are recruiting, I try to put a lot of emphasis on personal suitability. Psychic stability, how you behave, your attitude toward young people, your attitude toward criminality and abuse. Also, your attitude toward care and treatment of young people who are breaking norms, which I would say is at least as important as their formal education*. (Manager 4, residential home)

Managers also described how the wards physical environment was often shaped in certain ways to stimulate relationships with others, which enabled staff to be close to service users and care for them. Staff's ability to detect and de-escalate potentially violent situations was also mentioned by several managers as effective means to prevent violence, and managers were aware of the importance of staff acting swiftly and decisively in such situations.

*We were good at that, and the staff were very pleased with that, we quickly identifying what type of situation it was that triggered chains of events like this. When we removed this, we didn't have to deal with all the rest of it*. (Manager 1, child and adolescent psychiatric clinic)

#### Increasing Staff's Competence Regarding Safety and Handling of Violent Incidents

Another aspect of viewing staff as a resource was considering staff's continuous skills development; several ward managers reported that this was key to maintaining a safe care environment. Beyond the various broad efforts to increase staff competence, managers also gave examples of targeted education, such as the use of de-escalation techniques, physical techniques to meet violence, and security systems.

*We have something called TERMA here, and that is how you deal with threatening situations, and it involves treatment. It isn't just about learning how to wrestle, rather it's the whole treatment situation and being able to talk to the patient*. (Manager 1, forensic psychiatric clinic)

To maintain staff's level of competence in handling violent incidents, frequent exercises were implemented by several ward managers. There was a sense that these exercises should occur more regularly than was actually the case to prevent both staff and service users from injury when employing coercive measures, such as restraining service users. Taking time to reflect upon and deal with safety issues on the ward were, overall, considered important aspects of a ward manager's role, since the manager felt a responsibility to ensure both the safety of the patients and the staff's sense of being able to handle difficult situations.

#### Ensuring Sufficient Staffing Levels in Violent Situations

Several managers reported staff constellation and ensuring sufficient staffing levels in violent situations to be fundamental elements of primary and secondary prevention work within wards. Many ward managers were aware of their dependence on competent staff being in place and doing a good job, a job that would often come with some degree of personal sacrifice. Most ward managers expressed gratitude and admiration for their staff, and a fear of losing key individuals from their team. Some managers communicated their sense of resignation when confronting the difficulty of recruiting and not being able to retain key staff members, and pointed to a heavy work environment as a cause for this. Several ward managers described how staff came together and organized themselves around a service user in a collegially supportive manner to avoid potentially violent situations. Some managers also reported that staff stood by each other in any difficult situation, regardless of their differences or personal feelings toward each other.

*We make plans and try to organize as much as possible before the situation arises. Because often, they can be really angry, they can be in their room hitting furniture, trying to break things, but let them do that while we plan how to solve this. Situations rarely need to be solved in a hurry*. (Manager 2, forensic psychiatric clinic)

As a secondary prevention effort, staff were frequently transferred to and from different situations in which support was needed. Such efforts could mean calling in extra staff to stabilize the ward in general or, as was frequently reported, placing staff together with certain service users who were known to have a history of violent behavior.

*It could be, for example, that we have a vulnerable boy in the group, someone who is prone to violence or easily agitated, then we can have someone in place with that client all the time*. (Manager 3, residential home)

### Providing Information and Support to Staff

#### Enabling Meetings for Information and Support

One strategy for minimizing violence was prioritizing the information and communication aspects of care work. Several ward managers described this as securing access to the information necessary to make the right decisions and making sure that information was communicated to staff, and between staff, to create the safest possible care environment. In rounds and clinic meetings, ward managers conveyed information between wards and different professions. This exchange of information enabled a safe handling of problematic service users, as well as the allocation of necessary resources in the form of extra staff to prevent incidences of violence.

*Then there are treatment conferences where we specify and clarify, for each patient, strategies for dealing with difficult situations. Then we have development forums one hour every week where we mostly work with different ways of developing preventive work concerning coercive measures, although perhaps not so much from an ethical point of view*. (Manager 2, child and adolescent psychiatric clinic)

As illustrated in the quote above, managers discussed with staff how coercive measures can be carried out as respectfully and humanely as possible, but often discussed the matter as a practical problem rather than from an ethical perspective. These kinds of meetings also served the important purpose of proactively ensuring a safe work environment, such as preparing for an upcoming weekend where staffing levels would be lower and the risk of violent incidents would therefore be increased. Several ward managers reported that staff would typically raise important safety issues themselves and largely considered the variety of different forums for this purpose to be sufficient.

#### Organizing Debriefings

Tertiary prevention relied heavily on strategies that involved the frequent use of debriefing and supportive talks. The aim of this strategy was to validate staff's feelings and psychological wellbeing through sensitivity and communication after the occurrence of a violent situation. This strategy involved debriefing and guidance within the staff team, as well as the involvement of external resources to provide supportive talks when necessary.

*Let's say that I went down there now, and something has happened, then I would take the staff involved for a private talk and try to get a sense of what has happened and how the patient is doing. Do we need to take measures through occupational health care?* (Manager 2, child and adolescent psychiatric clinic)

The need for measures such as occupational health care was clear, and managers were committed to ensuring staff took this need seriously and having supportive talks. Overall, debriefing after violent incidents seemed to be an established practice, though this was not always appreciated by staff who oftentimes would consider the occurrence of violent incidents, or the possibility of this, as a natural part of everyday work on the ward and not something that demands a lot of discussion. Although the need for processing after different kinds of incidents was noted by most ward managers, some highlighted the difficulty of fitting in these types of discussions within the scope of everyday work. In these cases, the ward manager would need to actively create such a space, since this was not always planned.

*There aren't any real forums to solve this, so as a manager, one needs to be creative and actually create that kind of space. In a sense there is no natural spot for this to happen. No schedules are synced in that way*. (Manager 1, residential home)

After a violent incident, the need for support may differ between staff members, and many of the managers did not hesitate to involve external resources to facilitate the support process. Such resources included occupational health care personnel, internal therapists, and counselors. Generally, managers were also generous about giving staff time off, decreasing staff working hours, and giving staff time to recover after an incident. In some cases, staff were moved between wards. Ward managers' general impression was that staff are an invaluable resource whose psychological wellbeing needs to be maintained, even at a high economic cost. Many ward managers therefore made great effort to provide for the wellbeing of their staff both from a practical and an economical perspective.

## Discussion

The aim of this study was to document ward managers' strategies for prevention of violence in institutional care. Four such areas of prevention were identified, most of which influenced several levels of prevention. Violent incidents were seldom described in detail; rather, ward managers described their general strategies for preventing violence and managing critical incidents to minimize the impact on staff and service users. As revealed in previous studies (1–15), our findings indicate that institutional care settings are complex contexts in which many factors interact to form an unpredictable environment with a high risk of violent incidents and ethical dilemmas. Dilemmas about violence and coercion were, however, seldom discussed from an ethical perspective, and were more often considered as practical clinical problems or legal issues that needed to be solved appropriately. This may be interpreted as a lack of a functional “ethical grammar” within staff based on a common understanding regarding central ethical values that need to be respected within coercive care. It also requires a common language that can be used as a linguistic vehicle for common discussions in staff meetings, both on a general level and in connection with specific cases that need to be discussed in the working group. Needless to say, this also requires that specific time is set apart for ethical discussions, which is a task for the ward manager to arrange. In this way, we believe that there will be a collective development of higher ethical awareness regarding favorable human relations between staff and service users (49).

Our results indicate that primary prevention relies on ward managers promoting value awareness and strengthening relationships between service users and staff members, as well as acknowledging and nurturing staff's abilities and competence. This supports previous findings that violence prevention can be facilitated by ward managers' active spreading of positive attitudes and respect toward service users, as well as their support for staff's continuous training and setting examples of good behavior (30, 31). Some ward managers regretted the fact that praising staff in concrete situations was often hindered by their absence from work on the floor. Ward managers also supported staff by encouraging supportive relationships with service users, which can reduce violence (5, 20). Particularly within the care for children and adolescents, ward managers' active support in relationship-building was repeatedly highlighted as a key element to success in violence prevention, and was widely regarded as a foundation for violence prevention. In previous work, we interviewed staff and service users in the same wards as the ward managers interviewed in the present study. The staff at these same institutions also described the importance of relationships with service users (7, 46). Being close to service users gives staff members an opportunity to discover risks and it becomes easier to be successful in violence prevention if they already have a relationship with the service users. Interviews with service users revealed some good examples of staff who showed respect toward service users and who were open to communicating with them to create good relationships, but the opposite was also reported. In this study, ward managers worked with primary prevention by finding a balance between being a present and distant leader, as well as working with information flow and providing supportive talks. It was important for managers not to interfere too much with everyday work on the ward, and equally important to be available and supportive of staff. This illustrates the delicate balance between ward managers' faith that staff abide by written guidelines and to more specifically control the staff, which could underscore the difficulty of “forcing” such practice on staff (39). Some ward managers also noted that such guidelines need to be continuously updated to be practically useful.

Secondary prevention by ward managers was mainly centered around implementing non-coercive practices in encounters with service users, as well as acknowledging and strengthening staff's abilities and clinical competence. Ensuring sufficient staffing levels in any situation that called for extra resources was one way of ensuring that such situations would not escalate. Staff's early detection of potentially violent situations was pointed out as a principal means of de-escalating such situations, which supports previous findings that highlight the importance of risk assessment in decreasing violent incidents (7, 28, 32, 38). Similarly, applying de-escalation techniques has been found to promote violence prevention within a psychiatric inpatient care setting (26); this is also supported by the present study, whereby ward managers and staff made use of these techniques. In contrast to findings that staff are often unaware of such methods (6), many ward managers expressed an awareness of them and actively strived to promote them. While staff responses to violence have previously been described as needing improvement (5–7, 15), in this study, ward managers generally praised staff's abilities to react and respond to violent situations. Many employees in the participating wards had low education and the in-house training in dealing with violence and coercive measures was not ambitious in most of the wards. Therefore, employing staff with suitable personal characteristics was another way by ward managers to minimize violence and promoting the use of de-escalation. Many service users in the participating institutions reported being impressed by some staff members' ability to handle critical situations, but also that there were staff who provoked conflicts rather than mitigating them (5, 15). The staff in these institutions requested more education and training on how to deal with service users in critical situations, which was especially the case for staff members who did not have much experience. While inexperienced staff had more problems, staff believed that those with more experience would also benefit from more training (7, 46).

According to this study, the ward managers' focus of tertiary prevention was to provide information and support to staff. Although the importance of measures such as debriefing after violent situations was obvious to both managers and staff, it was often managers who insisted on this practice being maintained, which supports the view that this constitutes a successful leadership style (31). In our earlier research, effective communication between staff was emphasized as an essential part of violence prevention (7, 46), and, in the present study, ward managers seemed to be aware of its importance. Ward managers made many efforts to make sure that crucial information was communicated between both staff and other wards, particularly when there was a service user known to be potentially violent present on the ward, which called for additional staff resources to be allocated. Overall, ward managers viewed staff as a key resource in violence prevention, and therefore made efforts to support their health and wellbeing. This included frequent use of external resources such as internal therapists and counselors, as well as adjusting staff schedules and workload. These results support recommendations that management follow-ups with staff after encounters with service users behaving violently are important tertiary prevention measures (20, 43). However, previous research within the institutions included in this study has indicated that some staff members consider the support from ward managers after the occurrence of violent incidents to be inadequate (6, 7, 46). Staff described the main source of support to be their colleagues, and some were disappointed with the organization because they did not receive enough support from the management (6, 46). For staff, one of the main effects of violence was psychological problems, such as increased stress levels, sleep problems, and fear, which sometimes led to sick leave (6, 46). So, violence evokes a lot of emotions that staff have a hard time dealing with and ward managers do not seem to be aware that incidents can create problems long after they have occurred and affect the view of work and the professional role of staff members (6, 12, 14, 46).

The ward managers seem to have a high awareness of de-escalation and the work with secondary prevention, however, there seems to be a lack of evidence-based interventions to strengthen the primary prevention work. Regarding tertiary prevention, managers use de-briefing and supportive talks with staff, but in the case of service users, these follow-up talks were reported to be used more from a boundary setting perspective rather than debriefing the service user. So, there is a need to develop the prevention work in Sweden. There is the previously described course for staff, focusing mainly on de-escalation, but there is no policy at national or regional level to prevent violence and coercive measures with other evidence-based programs. This article is part of a larger project, “Prevention of violence in institutional care, aspects of ethics and safety”. This and previous publications (5–7, 9, 15, 46) show that services at times experience a conflict between values and safety, even if they do not express themselves with ethical terminology Our findings indicate that many staff and ward managers feel that they need more support and training on the issues of violence and coercion, but that managers are often busy dealing with daily difficulties. Ward managers in Sweden spend a lot of time on administration, most of them have a lack of administrative support and conduct many meetings outside their ward. This means that they rarely have time to reflect upon what a long-term sustainable prevention strategy could look like, and often don't have time enough to be present among staff and service users. The present results also reveal that managers wanted to promote a climate of reciprocal relationships between staff and service users, and that they argued for social justice by empathizing the importance of good care without using coercive measures. Managers considered creating safety to be a task for staff, and the service users were seen as recipients of safety measures rather than involving them in this process as partners, as recommended by World Health Organization (20). In Sweden, as in many other countries, policies that make use of evidence-based methods to reduce violence or coercive measures are currently lacking (18). A discussion on how care should relate to the Declaration on the Rights of Disabled Persons (35) and the Convention Against Torture and Other Cruel, Inhuman or Degrading Treatment or Punishment (34) is also largely lacking at the clinic level in Sweden.

## Strengths and Limitations

The primary aim of this study was to identify strategies used by ward managers to prevent violence in institutional care. By having interviewed 12 managers from six institutions, it was possible to identify a variety of strategies applied to the broad field of psychiatric and residential care, which is one of this study's strengths. However, this number of interviews is also a weakness, they are too few to be able to comment on differences and similarities between these institutions. Another strength is that comparisons were made between the researchers' coding and interpretations, and important decisions were taken with consensus. Limitations of the study include the fact that interviews were conducted in a single session, although the semi-structured interviews allowed for follow-up questions to be asked. Nonetheless, it would have been preferable to conduct follow-up interviews to clarify some responses. Also, the possibility that ward managers had an interest in presenting their work in an overly positive sense cannot be overlooked, which may have biased their responses. Fortunately, we interviewed staff and service users from the same clinics in previous studies, which made it possible for us to report on different stakeholders' perspectives in the discussion.

## Conclusions

Ward managers made use of several different strategies to minimize the level of violence in institutional care settings. Particularly in child and adolescent psychiatric clinics and special residential homes, managers found it challenging to create a balance between a high level of safety and good values toward service users. Strategies were applied to all three levels of prevention, and managers reported handling difficult situations in close cooperation with staff, whose importance for creating safe care environments was repeatedly underlined. The ward managers seem to have a high awareness of de-escalation and the work with secondary prevention, however, there is a need to develop the work with primary and tertiary prevention. Although managers described difficult ethical challenges surrounding violence and coercive measures, these were often described as practical problems. Thus, to enhance value awareness, there seems to be a need for a development of higher ethical awareness based on a common understanding regarding central ethical values to be respected in coercive care. Managers generally gave a more positive view about the prevention of violence than did staff and service users from the same institutions. Although the manager emphasized the competence of the staff, our other studies show that many staff members from the same institutions did not always feel listened to by their manager. In this study, no ward managers considered the service user group or user organizations as a resource in creating preventive strategies. To create safer care environments, there is a need to understand the perspectives on violence prevention of all stakeholders, including ward managers.

## Data Availability Statement

Data are not available since this could compromise the individual privacy of participants. The data are stored at the University Health Care Research Center, Region Örebro County, and may be available on request by other researchers, without undue reservations. Further enquires can be directed to the corresponding author.

## Ethics Statement

The studies involving human participants were reviewed and approved by the Ethical Review Board, Uppsala, Sweden, Reference Number 2014/112. The patients/participants provided their written informed consent to participate in this study.

## Author Contributions

VP-P, LK, and IE designed and planned the research project and provided valuable input to the manuscript. VP-P interviewed nine ward managers together with another member from the research group and made the last revision to the final manuscript. ML wrote the first draft of the manuscript. All authors participated in the process of coding, creating themes and subthemes, and accepted the final version of the manuscript.

## Funding

The study was funded by the Swedish Research Council for Health, Working Life and Welfare (2013-0389), The Swedish National Board of Institutional Care (2.6.1-221-2013) and Region Örebro County.

## Conflict of Interest

The authors declare that the research was conducted in the absence of any commercial or financial relationships that could be construed as a potential conflict of interest.

## Publisher's Note

All claims expressed in this article are solely those of the authors and do not necessarily represent those of their affiliated organizations, or those of the publisher, the editors and the reviewers. Any product that may be evaluated in this article, or claim that may be made by its manufacturer, is not guaranteed or endorsed by the publisher.
